# Emotional intelligence protects nurses against quiet quitting, turnover intention, and job burnout

**DOI:** 10.3934/publichealth.2024030

**Published:** 2024-04-25

**Authors:** Petros Galanis, Aglaia Katsiroumpa, Ioannis Moisoglou, Maria Kalogeropoulou, Parisis Gallos, Irene Vraka

**Affiliations:** 1 Clinical Epidemiology Laboratory, Faculty of Nursing, National and Kapodistrian University of Athens, Athens, Greece; 2 Faculty of Nursing, University of Thessaly, Larissa, Greece; 3 Department of Radiology, P & A Kyriakou Children's Hospital, Athens, Greece

**Keywords:** emotional intelligence, nurses, quiet quitting, turnover intention, job burnout

## Abstract

**Background:**

Emotional intelligence can improve nurses' interpersonal and coping skills, job performance, and resilience. However, there is a dearth in the literature on whether emotional intelligence affects levels of quiet quitting, turnover intention, and job burnout in nurses.

**Objective:**

We examined the relationship between emotional intelligence, quiet quitting, turnover intention, and job burnout.

**Methods:**

We conducted a cross-sectional study in Greece with a convenience sample of 992 nurses. We used the following valid tools to measure our study variables: the Trait Emotional Intelligence Questionnaire-Short Form, the Quiet Quitting Scale, and the single item burnout measure.

**Results:**

The mean age of our nurses was 42.2 years. After controlling for gender, age, work experience, shift work, and understaffed department, the multivariable linear regression models indicated significant negative relationships between emotional intelligence and quiet quitting, turnover intention, and job burnout. Specifically, self-control reduced detachment, lack of motivation, job burnout, and turnover intention. Moreover, emotionality reduced detachment, lack of motivation, and lack of initiative. Sociability reduced lack of initiative and lack of motivation, while well-being reduced lack of motivation, job burnout, and turnover intention.

**Conclusion:**

Emotional intelligence reduced quiet quitting, turnover intention, and job burnout in nurses. Therefore, nurse managers and policy-makers should apply interventions to optimize the emotional intelligence profiles of nurses.

## 1. Introduction

Emotional intelligence can be defined as the capacity of individuals to monitor their own feelings and others' feelings, and manage emotions in themselves and in their relationships [1]. There are two categories of attributes of emotional intelligence in professional nursing practice: personal and social [2]. Personal attributes refer to self-awareness and self-management, while social attributes refer to relationship management and social awareness. Since there is always an exchange of experiences, actions, and emotions between nurses and their patients, emotional intelligence plays a central role in all aspects of nursing practice [3], [4]. Thus, previous studies have showed the positive impact of nurses' emotional intelligence on patient outcomes, patient satisfaction, and quality service provisions [5]–[7]. On the other hand, low levels of emotional intelligence among nurses lead to burnout, work-related stress and work accidents, threaten the relationship between nurses and patients, increase patients' poor outcomes, reduce nurses' physical and mental well-being, and increase turnover intention [8]–[13].

Several systematic reviews and meta-analyses identified moderate to high levels of turnover intention and job burnout and reduced work satisfaction among nurses [14]–[20]. For example, a meta-analysis with 23,140 nurses found that the overall prevalence of turnover intention was 27.7% [17]. Also, a meta-analysis of 18,935 nurses found that the pooled prevalence of a lack of personal accomplishment, depersonalization, and emotional exhaustion was 15.2%, 12.6%, and 34.1%, respectively [15]. Moreover, working conditions worsened during the COVID-19 pandemic with nurses experiencing even greater levels of burnout, turnover intention, and dissatisfaction. The National Council of State Boards of Nursing reported that almost 100,000 nurses left their work during the pandemic in the USA, while 25% of all nurses intend to leave the workforce over the next five years [21]. Additionally, in the wake of the pandemic, the “quiet quitting” phenomenon started to threaten healthcare services, where employees offer the minimum of their capacity [22]. Interestingly, recent evidence suggests that a great percentage of nurses can be considered quiet quitters, and nurses acting as quiet quitters are found more often than other healthcare workers [23]. Quiet quitting has already been recognized as a significant threat for healthcare organizations, since the healthcare professionals may reduce their efforts to avoid job burnout, physical and mental outcomes, and occupational stress [22],[24]. Moreover, a recent study highlighted the negative consequences of quiet quitting for healthcare professionals and organizations as scholars found that quiet quitting increased turnover intention among nurses [25].

Several studies have investigated the relationship between emotional intelligence and work-related variables among nurses. In particular, high emotional intelligence has been linked to higher job satisfaction [9],[26],[27], higher work performance [27]–[31], higher professional success [32], less job burnout [9],[33], less occupational stress [31], and lower turnover intentions [34],[35]. In other words, high emotional intelligence is associated with the most beneficial work-related outcomes (i.e. work performance and job satisfaction), while low emotional intelligence is associated with the most negative work-related outcomes (i.e. job burnout, occupational stress, and turnover intention). Overall, literature suggests the protective effect of emotional intelligence against the adverse effects of the nursing profession.

In this context, emotional intelligence is an essential skill for nurses, and the impact of it on work-related variables should be examined in depth. There are only two studies, one in Spain and one in South Korea, that have investigated the relationship between emotional intelligence and job burnout [9],[33]. Moreover, another study in Taiwan investigated the relationship between emotional intelligence and turnover intention [35]. Additionally, there are no studies on whether emotional intelligence affects quiet quitting. Herein, our aim was to assess the impact of nurses' emotional intelligence on quiet quitting. Moreover, we examined the effect of emotional intelligence on turnover intention and job burnout among nurses.

## 2. Materials and methods

### 2.1. Design and participants

We performed a cross-sectional study during November 2023. We used a convenience sample and approached nurses in several ways. First, we used Google forms to create our online study questionnaire, and then we posted it on social media. Also, we collected data via paper and pencil to approach nurses that did not use social media frequently. Our nurses were required to work at least two years in a clinical setting and be Greek-speaking. We calculated the sample size of nurses as 652 by applying the following parameters: (a) a low effect size (*f^2^*=0.02) between our study variables, (b) nine independent variables, (c) 95% level of confidence, and (d) 5% margin of error.

### 2.2. Measures

Demographic variables included gender, age, work experience, shift work, and understaffed departments.

Moreover, our study included the followed instruments:

The Trait Emotional Intelligence Questionnaire-Short Form (TEIQue-SF) [36]: This 30-item measure assesses trait emotional intelligence. Participants respond on a 7-point Likert scale from 1 (completely disagree) to 7 (completely agree). TEIQue-SF provides scores on four factors (well-being, self-control, emotionality, sociability), and global trait emotional intelligence. Scores range on a scale from 1 to 7. Higher values indicate higher levels of emotional intelligence. We used the valid Greek version of the TEIQue-SF [37]. In our study, Cronbach's α for global trait emotional intelligence well-being, self-control, emotionality, and sociability was 0.882, 0.786, 0.639, 0.605, and 0.740, respectively.

The Quiet Quitting Scale (QQS) [38]: This 9-item measure evaluates three factors: detachment, lack of motivation, and lack of initiative. Scores range on a scale from 1 (low level of quiet quitting) to 5 (high level of quiet quitting). According to the developers of the QQS, a total score greater than 2.06 refers to quiet quitters [39]. The QQS has demonstrated very good psychometric properties [23]. Internal reliability analysis indicated acceptable Cronbach's α for QQS as well as for all three factors: 0.852 for the QQS; 0.761 for the factor “detachment”; 0.728 for the factor “lack of initiative”; and 0.747 for the factor “lack of motivation”.

The single-item burnout measure [40]: This one-item measure assesses job burnout on a scale from 0 (lower levels of burnout) to 10 (higher levels of burnout). A study including nurses in Greece has proven that the tool is valid [41].

The turnover intention scale [42]: A single question (“How often have you seriously considered leaving your current job?”) estimates turnover intention among workers. Answers are on a 6-point Likert scale from 1 (rarely) to 6 (extremely often). Workers with values ≥ 4 belong to a group with a high level of turnover intention, while workers with values from 0–3 belong to a group with a low level of turnover intention.

### 2.3. Ethical issues

We followed the Declaration of Helsinki to conduct our study [43]. Moreover, we had approval from the Ethics Committee of the National and Kapodistrian University of Athens, Faculty of Nursing (464, October 2023). We invited nurses to participate in an anonymous and voluntary way. We did not offer any compensation for participation.

### 2.4. Statistical analysis

Categorical variables are presented with numbers and percentages. Continuous variables are presented with mean, standard deviation, and median. We assessed the distribution of continuous variables with the Kolmogorov-Smirnov test, Q-Q plots, skewness values, and kurtosis values. Continuous variables followed normal distribution. Considering that quiet quitting and job burnout were continuous variables that followed normal distribution, we applied linear regression analysis. Also, considering that turnover intention was a dichotomous variable (high levels versus low levels), we applied logistic regression analysis. We performed univariate and multivariable regression analysis to eliminate confounding that was caused by the demographic variables. We presented unadjusted and adjusted coefficients beta, 95% confidence intervals (CI), *p*-values, and *R^2^* for the linear regression models. We presented odds ratios (OR), 95% CIs, *p*-values, and *R^2^* for the logistic regression models. *P*-values less than 0.05 were considered as statistically significant results. IBM SPSS 21.0 (IBM Corp. Released 2012. The IBM SPSS Statistics for Windows, Version 21.0. Armonk, NY: IBM Corp.) was used for statistical analysis.

## 3. Results

### 3.1. Participant characteristics

The detailed descriptive statistics for the demographic variables are shown in Table 1. The mean age of our nurses was 42.2 years.

**Table 1.** Demographic characteristics of nurses (*N* = 992).

**Characteristics**	***N***	**%**
Gender		
Males	94	9.5
Females	898	90.5
Age^a^	42.2	9.7
Years of work experience^a^	17.2	10.2
Shift work		
No	320	32.3
Yes	672	67.7
Understaffed department		
No	191	19.3
Yes	801	80.7

Note: ^a^ *mean*, standard deviation.

### 3.2. Study scales

Descriptive statistics for our study scales are shown in detail in Table 2. It can be seen that emotional intelligence, well-being, and emotionality were higher than self-control and sociability. Regarding quiet quitting, a lack of motivation was shown to be higher than a lack of initiative and detachment.

Among our nurses, 74.4% (*n* = 738) were considered as quiet quitters, while more than half of our nurses (51.6% [*n* = 512]) reported a high level of turnover intention.

**Table 2.** Descriptive statistics for our study scales (*N* = 992).

**Project**	**Mean**	**Standard deviation**	**Median**	**Kurtosis**	**Skewness**
**Emotional intelligence**	4.89	0.71	4.97	-0.17	-0.39
Well-being	5.25	0.99	5.33	0.48	-0.73
Self-control	4.65	0.90	4.67	-0.18	-0.24
Emotionality	4.94	0.78	5.12	0.29	-0.55
Sociability	4.45	1.01	4.50	-0.37	-0.25
**Quiet quitting**	2.55	0.74	2.44	0.30	0.60
Lack of motivation	3.02	0.94	3.00	-0.41	0.28
Detachment	2.35	0.82	2.25	0.63	0.73
Lack of initiative	2.52	0.92	2.33	-0.45	0.42
**Job burnout**	7.54	1.88	8.00	1.19	-0.98

### 3.3. Impact of emotional intelligence on quiet quitting, turnover intention, and job burnout

After controlling for demographic variables, the multivariable linear regression model indicated that self-control and emotionality reduced detachment. Moreover, emotionality and sociability reduced the lack of initiative, while sociability, self-control, emotionality, and well-being reduced the lack of motivation. Detailed results from the linear regression analyses are shown in Table 3.

**Table 3.** Relationship of emotional intelligence with quiet quitting (*N* = 992).

**Project**	**Lack of motivation**			**Lack of initiative**			**Detachment**		
	**Multivariable model^a,b^**			**Multivariable model^a,c^**			**Multivariable model^a,d^**		
	**Beta coefficient**	**95% CI for beta**	***P*-value**	**Beta coefficient**	**95% CI for beta**	***P*-value**	**Beta coefficient**	**95% CI for beta**	***P*-value**
**Well-being**	-0.19	-0.26 to -0.12	<0.001	-0.07	-0.14 to -0.004	0.038	-0.05	-0.11 to 0.01	0.116
**Self-control**	-0.09	-0.17 to -0.02	0.017	-0.07	-0.14 to 0.001	0.055	-0.16	-0.23 to -0.09	<0.001
**Emotionality**	-0.11	-0.19 to -0.03	0.007	-0.18	-0.26 to -0.10	<0.001	-0.18	-0.25 to -0.10	<0.001
**Sociability**	-0.10	-0.17 to -0.04	0.002	-0.21	-0.27 to -0.15	<0.001	-0.02	-0.08 to 0.04	0.512

Note: ^a^ The multivariable models were adjusted for the demographic variables: the *p*-value for ANOVA was <0.001 in all models; ^b^ *R^2^* = 12.6%; ^c^ *R^2^* = 20.7%; ^d^ *R^2^* = 16.2%; CI: confidence interval.

Similarly, we found a negative relationship between emotional intelligence and turnover intention. As shown in Table 4, the multivariable logistic regression model identified that the effect of well-being and self-control on turnover intention was significantly negative. Thus, nurses with higher levels of emotional intelligence were less probable to leave their work. *R^2^* for the final multivariable model was 14.9%.

**Table 4.** Relationship of emotional intelligence with turnover intention (*N* = 992).

**Project**	**Multivariable model^a^**		
	**Odds ratio**	**95% confidence interval for odds ratio**	***P*-value**
Well-being	0.79	0.66 to 0.94	0.007
Self-control	0.58	0.48 to 0.71	<0.001
Emotionality	0.92	0.75 to 1.13	0.420
Sociability	0.97	0.82 to 1.13	0.669

Note: ^a^ The multivariable model was adjusted for the demographic variables.

Additionally, we found a negative relationship between emotional intelligence and job burnout. In particular, we found that well-being and self-control were associated with decreased job burnout. *R^2^* for the final multivariable model was 14.2%. Detailed results from the linear regression analyses are presented in Table 5.

**Table 5.** Relationship of emotional intelligence with job burnout (*N* = 992).

**Project**	**Multivariable model^a^**		
	**Coefficient beta**	**95% confidence interval for beta**	***P*-value**
Well-being	-0.19	-0.34 to -0.06	0.006
Self-control	-0.16	-0.31 to -0.004	0.044
Emotionality	-0.04	-0.21 to 0.13	0.629
Sociability	-0.08	-0.21 to 0.05	0.202

Note: ^a^ The multivariable model was adjusted for the demographic variables.

## 4. Discussion

Our study was the first that examined the effect of nurses' emotional intelligence on quiet quitting. Additionally, since the literature is limited concerning the relationship between emotional intelligence, turnover intention, and job burnout among nurses, we examined these relationships. In our study, we assessed the direct effect of emotional intelligence after adjusting for several demographic variables. Our multivariable models demonstrated significant negative relationships between emotional intelligence, turnover intention, job burnout, and quiet quitting. In particular, our results suggested that emotional intelligence reduced quiet quitting, turnover intention, and job burnout in our nurses.

We found that nurses with higher levels of emotional intelligence experienced lower levels of quiet quitting. Although there is a dearth of literature regarding the relation between emotional intelligence and quiet quitting, evidence suggests a positive impact of emotional intelligence on several work-related variables. In particular, several studies showed that emotional intelligence had a positive effect on work performance [27]–[31], job satisfaction [9],[26],[27], organizational commitment [44], and clinical competence [45] among nurses. Since nurses are usually exposed to high levels of responsibility, stress, and emotional demands, emotional intelligence is a crucial tool to achieve the most beneficial outcomes. Emotional intelligence favors a good therapeutic relationship between nurses and patients, leading to greater adaptability and more positive attitudes, and protects nurses from occupational stress and burnout [46],[47]. In this context, nurses experience greater satisfaction with their work and improve their work performance [48]–[50]. A recent scoping review has identified that nurses are interested in enhancing their emotional intelligence because they recognize that emotional skills are necessary to better rationalize their experiences and emotions in the workplace [51]. Moreover, emotional intelligence improves nurses' ability to deeper understand their profession and show more spiritual maturity [52],[53]. Thus, more emotionally intelligent nurses can also acquire other intrinsic values, e.g., resilience, responsibility, self-awareness, self-management, and self-reflection [54]–[56].

In our study, more emotionally intelligent nurses experienced less burnout. Soto-Rubio et al. confirmed our findings by examining a sample of 125 nurses in Spain [9]. In a similar way, Kwon and Kim recognized the negative relationship between emotional intelligence and job burnout in a sample of nurses working at mental hospitals in South Korea [33]. A recent scoping review has emphasized the relevance of emotional intelligence in burnout prevention and emotional exhaustion [57]. Nurses with high emotional intelligence can create a positive work environment by incorporating spiritual motivation in their clinical practice [53],[56]. Moreover, emotionally intelligent nurses can identify the emotions of themselves, their patients, and their colleagues [51]. Thus, these nurses are more capable of coping with daily work challenges and adapting more effectively to work difficulties. In this context, nurses can handle excessive emotional and work-related expectations, thus avoiding high levels of job burnout [8],[9].

As a result of our study, emotional intelligence was a significant predictor of turnover intention. In particular, more emotionally intelligent nurses expressed lower intention to leave their job. Wang et al. confirmed our results as they found a significant negative relationship between emotional intelligence and turnover intention in a sample of nurses in Taiwan [35]. In a similar way, Al-Hamdan et al. examined the attitudes of 280 nurses in Jordan and found a positive correlation between emotional intelligence and intent to stay [34]. Our findings are in accordance with existing literature as studies found that leaders' emotional intelligence, as perceived by nurses, had a positive effect on turnover intention [58],[59]. Since turnover intention among healthcare workers has increased dramatically after the COVID-19 pandemic, identification of predictors of turnover intention is crucial to understanding the way that healthcare workers consider their future in the industry. For instance, one in four nurses in the USA intends to leave their work over the next five years [21]. Similarly, a recent survey in the USA revealed that 20% of physicians intend to leave primary care over the next three years [60].

Our study had some limitations. First, we cannot establish a causal relationship between our study variables since we performed a cross-sectional study. Longitudinal studies could add more information on our research questions. Second, we used a convenience sample and, thus, we cannot generalize our results. Future research should include random, stratified, and representative samples to improve our knowledge. Third, we used valid instruments to measure our study variables. However, our tools were self-reported and information bias is likely. Fourth, we performed our study in a sample of nurses in Greece with particular characteristics. Scholars should conduct studies in other cultural and contextual settings to shed more light on this issue. Finally, we constructed our multivariable models by eliminating several confounders, but several other variables can act as confounders. Thus, future studies should eliminate more confounders to extract more valid results.

## 5. Conclusions

Overall, our findings support the protective role of emotional intelligence against quiet quitting, turnover intention, and job burnout among nurses. However, considering the limitations of our study and the limited literature regarding our research questions, further studies should be conducted to get more valid results. Since the nursing profession demands mentally and physically strong nurses, high levels of emotional intelligence are crucial to maintain their mental and emotional well-being. Our study informs nurse managers and healthcare policymakers about the positive impact of emotional intelligence on work-related outcomes, such as turnover intention, quiet quitting, and job burnout. Therefore, nurse managers and healthcare policymakers should pay attention to the emotional intelligence of nurses by applying the appropriate interventions to develop emotional intelligence skills in nurses. For example, training programs focused on emotional intelligence, including interpersonal relationships, coping strategies, stress management, and relaxation techniques, may empower the psychological resources of nurses and improve their emotional intelligence. Appropriate emotional intelligence skills should be developed to improve nurses' commitment to their organization and retention.

## Use of AI tools declaration

The authors declare they have not used Artificial Intelligence (AI) tools in the creation of this article.
